# prevalence of addictions among students at the High School of The Health Sciences and Techniques of Sousse

**DOI:** 10.1192/j.eurpsy.2022.2141

**Published:** 2022-09-01

**Authors:** I. Betbout, B. Amemou, A. Ben Haouala, A. Sghaier, M. Sahli, F. Zaafrane, A. Mhalla, L. Gaha

**Affiliations:** 1 fattouma bourguiba hospital, Psychiatry, Psychiatry Research Laboratory Lr05es10 “vulnerability To Psychoses” Faculty Of Medicine Of Monastir, University Of Monastir, monastir, Tunisia; 2 High school of sciences and techniques of the health of Sousse, Psychiatry, sousse, Tunisia

## Abstract

**Introduction:**

Addiction isfrequent in youngsubjects, particularly in students, who are in contact with psychoactive substances such as drugs, tobacco, alcohol, and cannabis

**Objectives:**

The objectives of our study were to investigate the prevalence of addictions among ESSTSS students and to determine the factors associated with addictions.

**Methods:**

A descriptive correlational cross-sectional study was conducted at ESSTSS among 122 students for 2 months (March and April 2021). The data was collectedusing a questionnaire administered to the students.

**Results:**

There were 102 women and 20 men with an averageage of 20.96 years. theprevalence of drug use was 56.6% according to DAST-10, with addiction notedin 5.7% of cases. The prevalence of tobacco use was 35.3% according to the Fagerstörm test, with 23.3% of the students being highlyaddicted to tobacco. The prevalence of alcohol use was 29.5%, 35% for men, and 28.43% for womenaccording to the AUDIT, alcoholdependence was notedin 14.3% of men and 6.9% of women. The prevalence of cannabis use was 16.4% according to the CAST test, a high risk of dependence was observedin 20% of cases. The analytical study showedthat the factors associated with addiction were age, gender, year of study, and specialty

**Conclusions:**

The prevalence of substance use among health science students is significant and since the use of these substances has a detrimental effect on health itisbetter to understand the associated factors and this obliges us to establish appropriate preventive interventions

**Disclosure:**

No significant relationships.

